# Magnetic molecularly imprinted polymer coated with chitosan shell for enhanced controlled drug release

**DOI:** 10.1038/s41598-026-41273-0

**Published:** 2026-02-25

**Authors:** Najva Sadri, Mohammad Mazloum-Ardakani, Yvonne Joseph, Parvaneh Rahimi

**Affiliations:** 1 Department of Chemistry, Faculty of Science, Yazd University, Yazd, 89195- 741 Islamic Republic of Iran; 2 Institute of Nanoscale and Biobased Materials, Faculty of Materials Science and Technology, Technische Universität Bergakademie Freiberg, 09599 Freiberg, Germany

**Keywords:** Chitosan, Magnetic molecularly imprinted polymer, Controlled release, Drug delivery, pH-sensitive system, Chemistry, Drug discovery, Materials science

## Abstract

A pH-responsive chitosan-coated magnetic molecularly imprinted polymer (MMIP) was developed as a controlled-release drug carrier with enhanced selectivity, loading capacity, and release modulation. The carrier was constructed by synthesizing Fe_3_O_4_@SiO_2_ magnetic cores and forming a molecularly imprinted shell using 3-aminopropyltriethoxysilane (APTES) as the functional monomer. Imatinib (IMA**)**, a clinically important tyrosine kinase inhibitor, was used as both the model drug and the template to evaluate the carrier’s performance. To further enhance sustained and pH-responsive release, the MMIP was coated with a biocompatible chitosan (CS) layer after drug loading, yielding a hybrid core–shell nanocomposite (MMIP@CS). Fourier transform infrared spectroscopy (FT-IR), X-ray diffraction (XRD), vibrating sample magnetometry (VSM), microscopic images and dynamic light scattering (DLS) confirmed its structural integrity, magnetic properties, and well-controlled synthesis process. The system exhibited a high drug loading capacity (65.4%), reflecting strong molecular recognition within the imprinted cavities. The release studies at pH 7.4 and 5.5 demonstrated that the CS coating significantly improved release control over 96 h compared to the uncoated MMIP, enabling sustained and pH-dependent drug delivery. Kinetic modeling indicated that the Korsmeyer–Peppas model best described the release mechanism, suggesting a combined diffusion- and polymer-relaxation-controlled process. In vitro cytotoxicity assays further confirmed that IMA-MMIP@CS exhibited enhanced cytotoxicity against K562 cells while maintaining higher viability in normal PBMCs, highlighting its biocompatibility and therapeutic potential.

**Supplementary Information:**

The online version contains supplementary material available at 10.1038/s41598-026-41273-0.

## Introduction

Molecularly imprinted polymers (MIPs) have become an increasingly powerful platform in advanced drug delivery owing to their selective molecular recognition, structural stability, and tunable physicochemical properties^1–4^. These synthetic polymers are created through a process of polymerization, whereby a template molecule is combined with suitable monomers in the presence of a cross-linking agent. This results in the formation of specific cavities that are complementary in shape and functional groups to the target molecule or a group of structurally related molecules. This specific property of selective binding has the potential to significantly enhance the efficacy of drug delivery systems. Moreover, MIPs demonstrate stability, versatility, and the capacity to be engineered for responsiveness to diverse stimuli, including light, temperature, magnetic fields, and tumor microenvironment factors such as pH, reducing agents, enzymes, and reactive oxygen species. This responsiveness enables the controlled release of therapeutic agents at specific sites, enhancing therapeutic efficacy while reducing adverse effects^1^. These features position MIPs as a promising option for smart drug delivery technologies.

Among these innovations, the utilization of magnetic stimuli and the advancement of MIPs responsive to magnetic fields, achieved by combining their selective binding properties with iron oxide magnetic nanoparticles (NPs), particularly Fe_3_O_4_, have resulted in the creation of highly versatile magnetic MIP (MMIP) hybrid systems. These MMIPs provide enhanced precision in drug targeting and controlled release, significantly advancing the potential for more effective and targeted therapies^5,6^. Nevertheless, the utilization of bare Fe_3_O_4_ NPs for the development of MMIPs presents certain challenges. Due to their considerable surface area and hydrophobic nature, these nanoparticles are susceptible to substantial agglomeration under the influence of a magnetic field. Additionally, they tend to form granule clusters with plasma proteins, which can result in their rapid clearance from the circulatory system by the liver and kidneys^7–9^. The surface modification of Fe_3_O_4_ NPs with biopolymers^10^, synthetic polymers^11^, silica (SiO_2_)^12^, SiO_2_-gold^13^ or other organic compounds like azo compounds (R − N = N − R′)^14^ can effectively overcome the aforementioned limitations, thereby improving their biocompatibility and circulation time in the bloodstream. This, in turn, enhances the efficiency and precision of MMIP-based drug delivery systems. Among these modifications, the SiO_2_ coating is particularly effective due to its multiple advantages^15^. It serves as a protective layer that prevents agglomeration, enhances the biocompatibility of Fe_3_O_4_ NPs, and provides surface functional groups for further chemical modifications^16,17^. These features make the SiO_2_ coating an ideal choice for use in MMIP-based drug delivery systems^7,9,12^.

On the other hand, pH-responsive MIPs represent an intelligent approach to targeted drug delivery, leveraging the acidic environment characteristic of cancerous tumors (pH ~ 5.5) in comparison to that of normal tissues (pH ~ 7.4)^18,19^. The coating of MMIPs with pH-sensitive materials creates a promising multi-stimuli platform, enabling precise and efficient drug release specifically within tumor sites. This dual-response system, which combines magnetic guidance and pH sensitivity, enhances more efficiently the targeted delivery and controlled release of therapeutics. In this context, CS has attracted considerable attention over the past decade as a biocompatible, biodegradable, and pH-responsive biopolymer in controlled drug delivery systems^20–23^. The appeal of CS lies in the presence of hydroxyl (–OH) and amine (–NH₂) functional groups along its polymer chains, which allow it to respond to pH variations. At pH levels below 6, the protonation of CS’s amino groups facilitates drug release more effectively in acidic environments, making it an ideal candidate for targeted delivery within tumor microenvironments^24–27^.

Imatinib (IMA) is a highly potent pyrimidine derivative in the class of tyrosine kinase inhibitors that is widely used to treat various malignancies including leukemia^28^, tumors^29^, and other malignancies^30,31^. It plays a vital role in suppressing the BCR-ABL fusion protein, a key driver of chronic myeloid leukemia (CML). Despite its efficacy, conventional administration of IMA requires high dosages, often causing severe side effects and contributing to drug resistance^32,33^. Drug delivery technologies therefore offer a promising means of improving the therapeutic index of IMA by enhancing its targeted transport and reducing systemic toxicity^34^, and numerous nanoscale systems have been explored for this purpose, including microemulsions^35^, microspheres^36^, liposome^37^, micelle^38^, and nanoparticle^39^.

In this study, we developed a hybrid system based on chitosan-coated magnetic molecularly imprinted polymers (IMA-MMIP@CS) for the loading and controlled release of IMA a drug model. The synthesized Fe_3_O_4_ NPs, modified with a SiO_2_ shell (Fe_3_O_4_@SiO_2_), functioned as the core structure. Onto this core, an MIP layer was constructed using APTES as the functional monomer, tetraethyl orthosilicate (TEOS) as the cross-linker, and IMA as the template molecule. In fact, the amino group (-NH₂) of APTES as a functional monomer can form hydrogen bonds with the oxygen of IMA’s amide carbonyl and the nitrogen atoms in the pyridine or piperazine rings of IMA. Finally, the resulting magnetic imatinib-imprinted polymer (IMA-MMIP) was coated with CS and referred to as IMA-MMIP@CS. The structure and essential properties of this hybrid system were characterized using a range of analytical techniques, FT-IR, XRD, Energy Dispersive Spectroscopy (EDS), VSM, field emission scanning electron microscopy (FE-SEM) transmission electron microscopy (TEM), DLS, and zeta potential measurements. In order to ascertain the function of CS in the loading and release of IMA, two drug delivery systems, IMA-MMIP and IMA-MMIP@CS, were evaluated at two distinct pH levels (5.5 and 7.4). These levels were selected to reflect conditions that are analogous to those present in tumor microenvironments and normal tissues, respectively. Furthermore, the drug release kinetics were analyzed by fitting the experimental data to a number of mathematical models, including the Zero-order, First-order, Higuchi, Korsmeyer-Peppas, and Hixson-Crowell models.

## Materials and methods

### Chemicals and reagents

Imatinib mesylate was supplied by Baran Chemical and Pharmaceutical Company (Tehran, Iran). TEOS, APTES, glutaraldehyde solution (25%), ammonia solution (28%), medium molecular weight chitosan (200–800 cps), 3-(4,5-dimethyl-thiazol-2-yl)-2,5-diphenyl tetrazolium bromide (MTT), Dulbecco’s modified Eagle medium (DMEM), fetal bovine serum (FBS) and dimethyl sulfoxide (DMSO) were obtained from Sigma-Aldrich. Sodium chloride (NaCl), potassium chloride (KCl), disodium phosphate (Na₂HPO₄), sodium dihydrogen phosphate (NaH₂PO₄), ferric chloride hexahydrate (FeCl₃·6 H₂O), ferrous chloride tetrahydrate (FeCl₂·4 H₂O), ammonia solution, ethanol, methanol, acetic acid, hydrochloric acid (HCl), and sodium hydroxide (NaOH) were purchased from Merck (Darmstadt, Germany). A dialysis membrane with a molecular weight cut-off of 12 kDa was also sourced from Sigma-Aldrich.

### Instruments

The ultraviolet-visible (UV-Vis) spectrum of the drug and MIPs was measured at room temperature using an OPTIZEN 3220 UV spectrophotometer from Korea. The morphology of the particles was evaluated using transmission electron microscopy (model Philips EM 208 S, Netherlands) and field emission scanning electron microscopy (model TESCAN MIRA2, Czech Republic) to obtain TEM and FE-SEM images. The particle size, size distribution, and zeta potential of the IMA-MMIP@CS in phosphate-buffered saline (PBS) media were assessed using a Horiba Zeta potential analyzer from the UK. VSM were studied using a vibrating sample magnetometer (Meghnatis Daghigh Kavir Co., Kashan, Iran) at room temperature. XRD of nanoparticles was recorded using an X-ray diffractometer (Philips PW 1730/10). FT-IR analysis was carried out using KBr discs in the region of 4000–400 cm^− 1^ by BRUKER EQUINOX 55 single beam spectrometer. EDS (model EM8000F, China) was used to identify and quantify the elements present in IMA-MMIP@CS.

### Synthesis of iron oxide @ silica core/shell (Fe_3_O_4_@SiO_2_)

Magnetic Fe₃O₄ NPs were fabricated through a conventional co-precipitation method^40^. In this process, 1.625 g (8 mmol) of FeCl_2_·4H_2_O and 4.43 g (16 mmol) of FeCl_3_·6H_2_O were dissolved in 190 mL of deionized water (DW) at room temperature with mechanical stirring at 600 rpm. Next, 15 mL of 28% ammonia was added to the salt solution, leading to the appearance of a black Fe_3_O_4_. After stirring for 10 min, the precipitate was separated from the solution using a magnet and washed three times with ethanol and DW.

Fe₃O₄ NPs were silica-coated via a modified Stöber process to enhance the stability and enable additional functionalisation. 10 mg of the freshly prepared Fe₃O₄ particles were ultrasonically dispersed in 120 mL of a mixture of ethanol and DW. Then, 6 mL of ammonia solution (28%) was added and the mixed dispersion was vigorously stirred (700 rpm) for 10 min at room temperature. The stirring speed was then decreased to 300 rpm, and 0.3 mL of TEOS was added dropwise into the solution for 15 min. Finally, the dispersion stood for 12 h for continuous hydrolysis of TEOS, resulting in the formation of core−shell Fe₃O₄ @SiO₂ NPs. Under identical conditions, three independent batches of Fe₃O₄@SiO₂ NPs were synthesized to evaluate the reproducibility of the process and the size uniformity of the resulting NPs. The Fe₃O₄@SiO₂ nanoparticles were magnetically separated, washed repeatedly with ethanol and deionized water, and dried under ambient conditions^41^.

### Synthesis of MMIP

The MMIP was fabricated through a stepwise procedure. Initially, IMA (5 mg) and APTES (23 µL) were dissolved in 30 mL of ethanol and stirred at 500 rpm for 30 min, so that effective interaction between the monomer and template could occur. Subsequently, TEOS (46 µL) was introduced, and the mixture was stirred again for 20 min at 500 rpm. Afterwards, Fe₃O₄@SiO₂ NPs (20 mg) and 1 mL of 0.01 M HCl solution were added, and the reaction was maintained under continuous stirring at 500 rpm for 12 h. The resulting MMIP was magnetically separated and dried at 60 °C for 8 h.

**Fig. 1:**
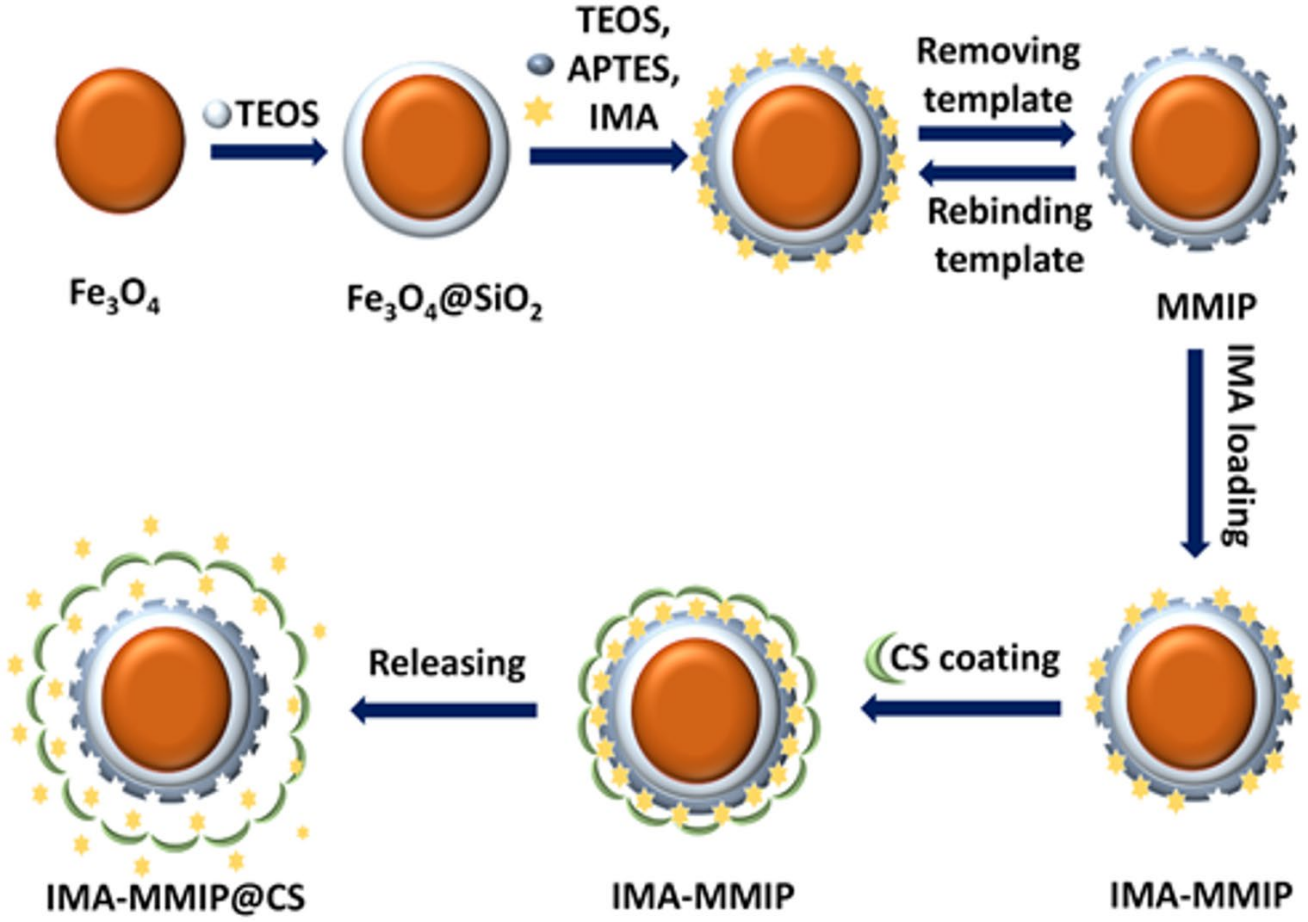
Schematic illustration of the stepwise synthesis of IMA-MMIP@CS, including magnetic core formation, silica coating, IMA molecular imprinting and loading, chitosan (CS) coating, and IMA release from the nanocarrier.

The removal of the IMA template from the MMIP structure was achieved by sequentially washing the particles with 50 mL of a methanol/acetic acid solution (9:1, v/v). This was done three times, with each wash carried out under continuous stirring at 500 rpm for 1 h. Successful extraction of the template molecules was verified through UV–Vis spectrophotometric analysis. Following this step, the particles were rinsed thoroughly with pure methanol to remove any remaining acetic acid, and subsequently dried at 60 °C for an additional 8 h. For comparison purposes, magnetic non-imprinted polymer (MNIP) particles were synthesized using the same experimental conditions, except that the IMA template was omitted from the formulation^42^. Figure 1 schematically represents the synthesis of IMA-MMIP@CS and highlights the steps involved in the loading and controlled release of IMA.

### Synthesis of CS decorated IMA-MMIP (IMA-MMIP@CS)

To prepare IMA-MMIP@CS, 50 mg of IMA-MMIP was dispersed in 30 mL of DW using ultrasonic vibration for 5 min. Next, 10 mL of 25% glutaraldehyde was added and stirred for 10 min at 500 rpm. Then, 10 mL of CS solution (1% w/v in 1% acetic acid) was gradually added and stirred for 8 h. The resulting IMA-MMIP@CS precipitation was magnetically separated and dried at 60 °C for 8 h^43^. Typical yields of Fe₃O₄@SiO₂ were 85 ± 4% per batch (*n* = 3), and the overall yield of IMA-MMIP@CS after template removal and coating was 78 ± 5% per batch (*n* = 3), indicating good batch-to-batch reproducibility.

### Drug loading onto MMIP

To load IMA onto the proposed MMIP, 10 mg of MMIP was dispersed in 10 ml ethanol/DW solution (1:1, v/v) containing various concentrations of IMA (0.2, 0.4, 0.6, 0.8, 1.0 and 1.2 mg/mL). The mixture was stirred for 12 h at room temperature under the protection of light. The IMA-loaded MMIP (IMA-MMIP) was then separated using a magnet and washed with DW to remove any unbound IMA. The supernatant was then analyzed using a UV-Vis spectrophotometer to calculate the drug loading capacity (DLC) and drug loading efficiency (DLE) of IMA (λ_max_ = 255 nm) using the calibration curve (Figure S1A) based on Eqs. (1) and (2) respectively^44^:

1 $$\:\mathrm{D}\mathrm{L}\mathrm{C}\:\left(\mathrm{m}\mathrm{g}/\mathrm{g}\right)=\frac{\mathrm{v}({\mathrm{C}}_{0}-{\mathrm{C}}_{\mathrm{f}})}{\mathrm{m}}$$

2 $$\:\mathrm{D}\mathrm{L}\mathrm{E}\:\left({\%}\right)=\:\frac{{\mathrm{C}}_{0}-{\mathrm{C}}_{\mathrm{f}}}{{\mathrm{C}}_{0}}$$

Here, V (ml) represents the volume of the solution, C_0_ (mg/ml) represents the initial drug concentration, C_f_ (mg/ml) represents the drug concentration in the supernatant solutions, and m (g) represents the applied weight of the MIP. The drug loading process was also carried out for MNIP using the optimized drug amount. The complete UV–Vis calibration data, raw drug loading calculations, and template leaching results are provided in the Supplementary Information (Tables S2–S3 and Figure S1). A comparative UV–Vis analysis of MMIP, IMA-MMIP, and drug-loaded MNIP (IMA-MNIP) is presented in Figure S2.

The imprinting performance of the MIPs was evaluated by calculating the imprinting factor (IF)^45^, expressed as:

3 $$\:\mathrm{I}\mathrm{F}=\:\frac{{\mathrm{D}\mathrm{L}\mathrm{C}}_{\mathrm{M}\mathrm{I}\mathrm{P}}}{{\mathrm{D}\mathrm{L}\mathrm{C}}_{\mathrm{N}\mathrm{I}\mathrm{P}}}$$

Where $\:{\mathrm{D}\mathrm{L}\mathrm{C}}_{\mathrm{M}\mathrm{I}\mathrm{P}}$ (mg/g) and $\:{\mathrm{D}\mathrm{L}\mathrm{C}}_{\mathrm{N}\mathrm{I}\mathrm{P}}$ (mg/g) represent the amounts of the template molecule bound to the MIP and the NIP, respectively.

### Drug release experiments

This study aimed to investigate the release of IMA from IMA-MMIP and IMA-MMIP@CS at 37 ˚C under two different pH conditions (pH = 5.5 and 7.4) using PBS solution. Two suspensions of IMA-MMIP and IMA-MMIP@CS (5.0 mg/mL) were prepared, and 1 mL of each samples were mixed with 1 mL of each buffer solution. Two dialysis bags (molecular weight cutoff 12 kDa) were filled with the mixture and placed in 5 mL of the same buffer solution. The solutions were shaken at 37 ˚C. At each release measurement, 1 mL of solution was removed from the tube and replaced with 1 mL of fresh buffer. The concentration of IMA in the collected samples was quantified using a UV–Vis spectrophotometer at 255 nm, based on calibration curves shown in Figure S1 (B and C). The UV–Vis spectra illustrating drug release from nanocarriers under two pH conditions are presented in Figure S3. The cumulative release rate (CR%), was calculated by the following equation^46^:

4 $$\:\mathrm{C}\mathrm{R}\left({\%}\right)=\frac{\mathrm{V}{\mathrm{C}}_{\mathrm{n}}+\mathrm{v}\sum\:{\mathrm{C}}_{(\mathrm{n}-1)}}{{\mathrm{W}}_{0}}\times\:100$$

Here W_0_ represents the weight of drug in carrier, C_n_ and C_*n*−1_ represent the drug concentration in the solution collected at (n) and (n-1) times respectively, V is the total detected volume, and v stands for the withdrawn and replaced fresh buffer solution.

### Drug release kinetics

The experimental drug release data were analyzed by applying five different kinetic models, namely Zero-order, First-order, Higuchi, Korsmeyer–Peppas, and Hixson–Crowell (summarized in Table 1). In these equations Q_t_ refers to the cumulative amount of drug released at a given time t while Q_0_ represents the initial amount released, generally taken as zero. The ratio $\:{\mathrm{M}}_{\mathrm{t}}/{\mathrm{M}}_{{\infty\:}}$ indicates the fraction of the total drug released at time t, and n is the release exponent that characterizes the release mechanism. The constants k_0_, k_1_, k_H_, k_KP_, and k_HC_, corresponding to Zero-order, First-order, Korsmeyer-Peppas, Higuchi, and Hixson-Crowell models, respectively^47–51^. The appropriate kinetic model was identified through curve.

**Table 1:** Mathematical models applied to drug release kinetics fitting and correlation coefficient (R^2^) comparisons.

Model	Equations	Applications
Zero-order	$\:{\mathrm{Q}}_{\mathrm{t}}-{\mathrm{Q}}_{0}={\mathrm{k}}_{0}.\mathrm{t}$	Used for osmotic pump systems, transdermal delivery devices, and matrix tablets formulated for drugs with low solubility, including coated dosage forms.
First-order	$\:{\mathrm{Q}}_{\mathrm{t}}-{\mathrm{Q}}_{0}={\mathrm{e}}^{-{\mathrm{k}}_{1}.\mathrm{t}}$	Suitable for systems where drug release is governed by matrix diffusion and dissolution, such as sustained-release formulations.
Higuchi	$\:{\mathrm{Q}}_{\mathrm{t}}={\mathrm{k}}_{\mathrm{H}}{.\mathrm{t}}^{0.5}$	Applied to matrix-based systems, transdermal therapeutic patches, and topical gel preparations.
Korsmeyer _ Peppas	$\:{\mathrm{M}}_{\mathrm{t}}/{\mathrm{M}}_{{\infty\:}}={\mathrm{k}}_{\mathrm{K}\mathrm{P}}{.\mathrm{t}}^{\mathrm{n}}$	Describes drug release from polymeric carriers and various modified-release dosage forms.
Hixson-Crowell	$\:{(1-{\mathrm{M}}_{\mathrm{t}}/{\mathrm{M}}_{{\infty\:}})}^{1/3}={\mathrm{k}}_{\mathrm{H}\mathrm{C}}.\mathrm{t}$	Employed for erodible matrix formulations where surface area and diameter change during release.

### In vitro cytotoxicity evaluation

The in vitro cytotoxicity of the nanocarriers, free IMA, and IMA-loaded nanocarriers was evaluated using the MTT colorimetric assay. The human chronic myelogenous leukemia cell line K562 was selected as the cancer model, while peripheral blood mononuclear cells (PBMCs) were used as normal human cells. The experiments involving the K562 cell line and PBMC samples were conducted with technical assistance in a private laboratory facility. K562 is a well-established, commercially available human cell line. PBMC samples were handled according to standard laboratory procedures using anonymized samples. Both cell types were cultured in DMEM supplemented with 5% FBS, 100 µg/mL penicillin G, and 100 µg/mL streptomycin, and maintained at 37 °C in a humidified atmosphere containing 5% CO₂. K562 cells and PBMCs were seeded separately into 96-well plates at a density of 1 × 10⁴ cells per well and allowed to stabilize prior to treatment. Subsequently, UV-sterilized samples were then added at predetermined concentrations. IMA was administered either in its free form or encapsulated within nanocarriers (IMA-MMIP and IMA-MMIP@CS) at final concentrations of 0.5, 1.0, 5.0, 10.0, 25.0, and 50.0 µg/mL.

Following incubation periods of 24, 48, and 72 h, cell viability was quantified by MTT assay. The culture medium was removed, and the cells were washed with PBS. Thereafter, 100 µL of MTT solution (0.5 mg/mL) was added to each well and the plates were incubated for an additional 4 h to allow the formation of formazan crystals. The supernatant was then discarded, and 200 µL of DMSO was added to dissolve the resulting crystals completely.

Absorbance was measured using a microplate reader, and cell viability was expressed as a percentage relative to untreated control cells according to the following equation:

5 $$\:\mathrm{C}\mathrm{e}\mathrm{l}\mathrm{l}\:\mathrm{v}\mathrm{i}\mathrm{a}\mathrm{b}\mathrm{i}\mathrm{l}\mathrm{i}\mathrm{t}\mathrm{y}\:\left({\%}\right)=\frac{\mathrm{m}\mathrm{e}\mathrm{a}\mathrm{n}\:\mathrm{o}\mathrm{f}\:\mathrm{a}\mathrm{b}\mathrm{s}.\:\mathrm{v}\mathrm{a}\mathrm{l}\mathrm{u}\mathrm{e}\:\mathrm{o}\mathrm{f}\:\mathrm{t}\mathrm{r}\mathrm{e}\mathrm{a}\mathrm{t}\mathrm{m}\mathrm{e}\mathrm{n}\mathrm{t}\:\mathrm{s}\mathrm{a}\mathrm{m}\mathrm{p}\mathrm{l}\mathrm{e}}{\mathrm{m}\mathrm{e}\mathrm{a}\mathrm{n}\:\mathrm{o}\mathrm{f}\:\mathrm{a}\mathrm{b}\mathrm{s}.\:\mathrm{v}\mathrm{a}\mathrm{l}\mathrm{u}\mathrm{e}\:\mathrm{o}\mathrm{f}\:\mathrm{c}\mathrm{o}\mathrm{n}\mathrm{t}\mathrm{r}\mathrm{o}\mathrm{l}\:\mathrm{s}\mathrm{a}\mathrm{m}\mathrm{p}\mathrm{l}\mathrm{e}}\times\:100$$

All the experiments were performed in triplicate to acquire the accurate results.

## Results and discussion

### FT-IR spectra and XRD analysis

**Fig. 2:**
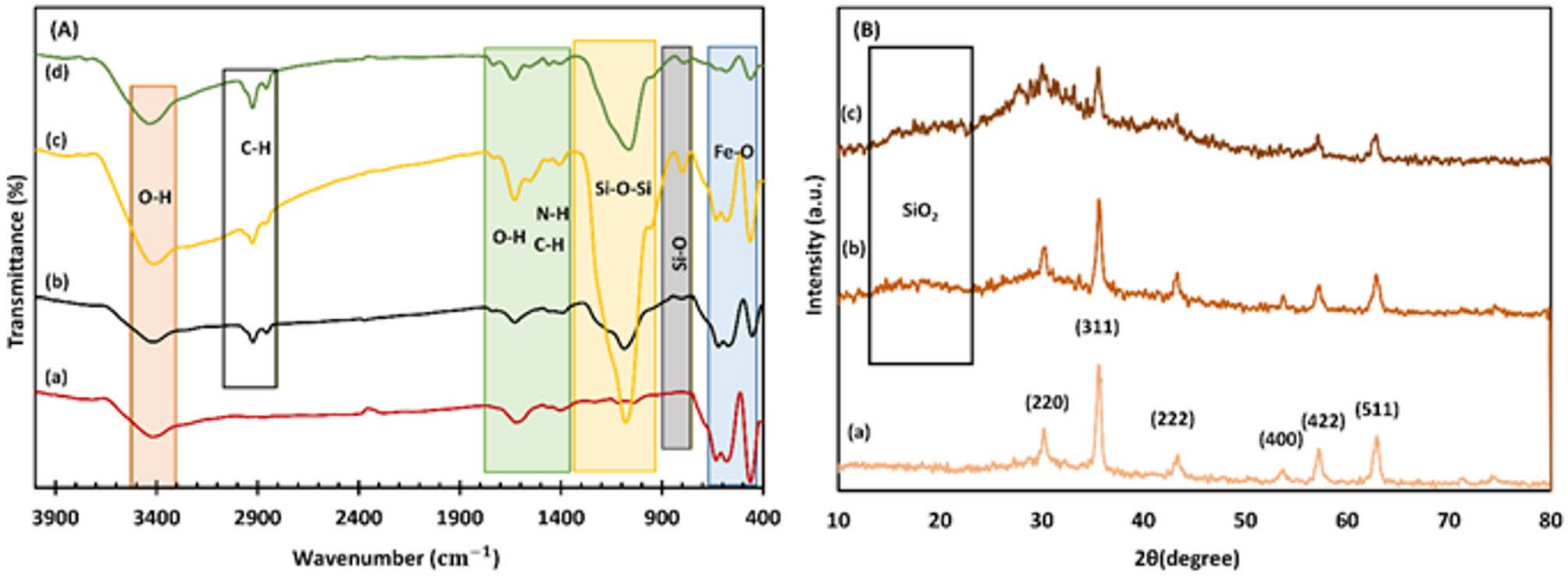
(**A**) FT-IR spectra of (a) Fe_3_O_4_, (b) Fe_3_O_4_@SiO_2_, (c) MMIP, and (d) IMA-MMIP@CS. (**B**) XRD pattern of (a) Fe_3_O_4_, (b) Fe_3_O_4_@SiO_2_ and (c) IMA-MMIP@CS.

The FT-IR spectra of Fe_3_O_4_, Fe_3_O_4_@SiO_2_, MMIP, and IMA-MMIP@CS were analyzed, as illustrated in Fig. 2A. The synthesized Fe_3_O_4_ NPs were confirmed by the presence of characteristic absorption peaks at 572 and 632 cm^− 1^, which correspond to the Fe-O stretching vibration. Additionally, the absorption band observed at 459 cm^− 1^ is attributed to the Fe-O vibrations at the tetrahedral and octahedral sites within the Fe_3_O_4_ structure^51^. The band at 1620 and 3402 cm^− 1^ are due to water molecules adsorbed on the magnetite surface and O-H bending vibrations, respectively^52^. The successful coating of SiO_2_ layers onto Fe_3_O_4_ particles is evident from the absorption band at 1083 cm^− 1^, corresponding to the asymmetrical stretching vibrations of Si–O–Si. Two weaker absorption bands at 954 and 796 cm^− 1^ are due to the symmetrical stretching vibration of Si–O–Si, and the stretching vibration of Si–O, respectively^53–56^. The spectra of MMIP and Fe_3_O_4_@SiO_2_ show the same characteristic bands. However, the extreme increase in the intensity of the Si–O–Si peak in MMIP indicates the successful polymerization of APTES on the previous SiO_2_ layer. Moreover, the spectrums of MMIP and IMA-MMIP@CS display characteristic absorption bands at 1400, 1550, 2848, and 2923 cm^− 1^, confirming the presence of the N–H and C–H group after drug and CS loading^55,57–59^.

XRD analysis was utilized to investigate the nanostructure of the Fe_3_O_4_, Fe_3_O_4_@SiO_2_ and IMA-MIP@CS. The sharp diffraction peaks of Fe_3_O_4_ observed at 2θ values of 30.5°, 35.6°, 43.3°, 53.6°, 57.2°, and 62.9° (as shown in Fig. 2B (a)) correspond to the crystal planes (220), (311), (400), (422), (511), and (440), respectively. These peak positions are in perfect agreement with the standard database of magnetite in the JCPDS-International Center (JCPDS card: 19–0629). The same peaks in Fig. 2B (b) and (c) further confirm the unchanged magnetite structure and the stability of Fe_3_O_4_ phase^60,61^. Compared with Fig. 2B (a), the intensity of the characteristic diffraction peak of Fe_3_O_4_ in Fig. 2B (b) has decreased, and a new broad peak appeared at 2θ = 18–25^◦^, indicating amorphous SiO_2_. This demonstrates that SiO_2_ has been successfully coated onto the surface of Fe_3_O_4_ NPs^61^. In Fig. 2B (c), compared to Fig. 2B (b), the peak positions of SiO_2_ and Fe_3_O_4_ remain unchanged. However, the corresponding peak intensities have slightly decreased, suggesting the successfully deposition of both MIP and CS layer onto the surface of Fe_3_O_4_@SiO_2_.

### Morphological characterization and EDS analysis

**Fig. 3:**
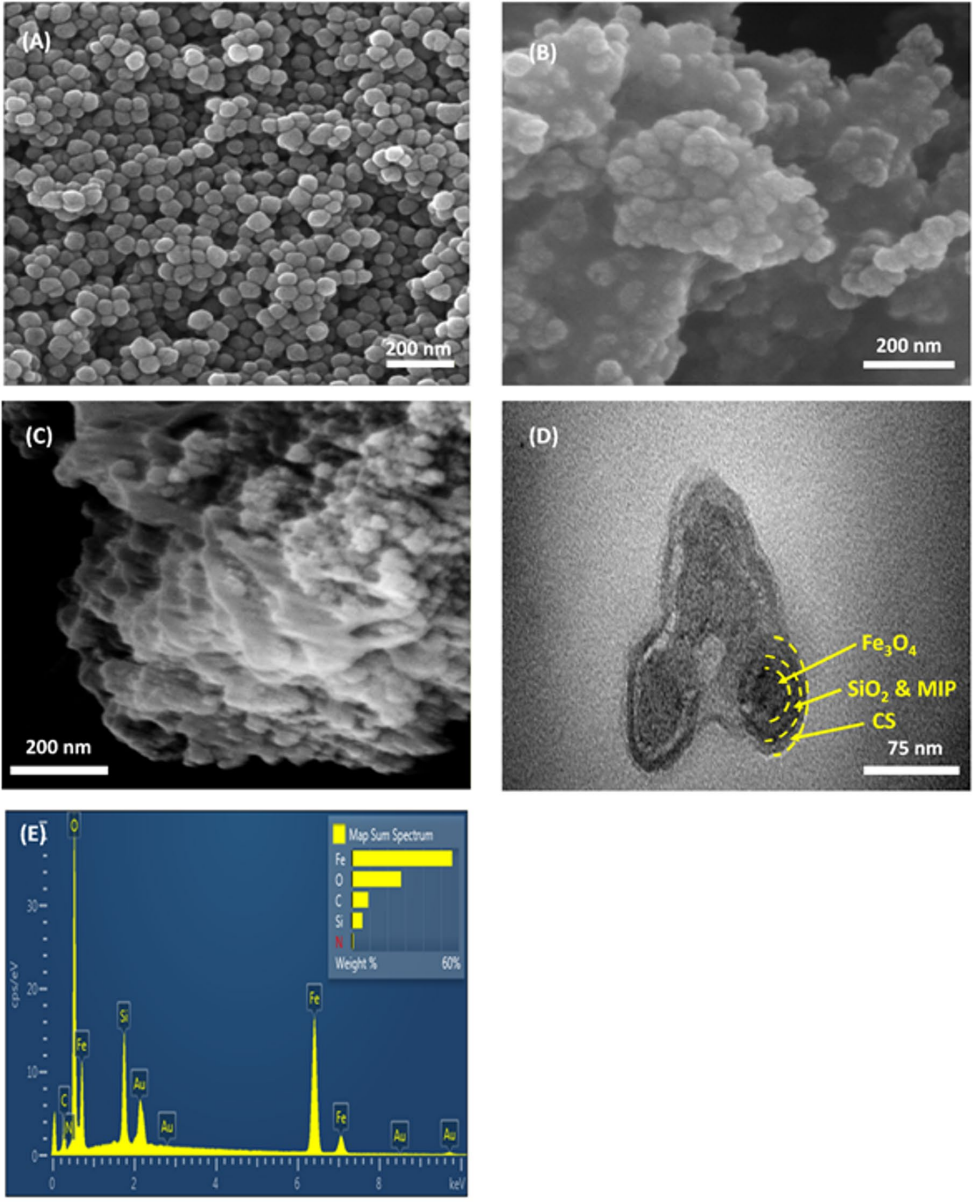
FE-SEM images of **A**) Fe_3_O_4_ NPs, **B**) Fe_3_O_4_@SiO_2_ NPs and **C**) IMA-MMIP@CS. **D**) TEM image of IMA-MMIP@CS. **E**) EDS spectrum of IMA-MMIP@CS.

The morphology and structure of the synthesized materials were characterized using FE-SEM and TEM images. Figure 3A presents a FE-SEM image of Fe_3_O_4_ NPs. These particles exhibit a spherical-like morphology, with diameters ranging between 40 nm and 50 nm. The uniformity of particle size and shape is clearly visible, indicating a well-controlled synthesis process. After coating with the SiO_2_ layer, the core-shell Fe_3_O_4_@SiO_2_ NPs retained the spherical shape of the original Fe_3_O_4_ NPs, but their size was significantly larger than that of the uncoated Fe_3_O_4_ NPs, as shown in Fig. 3B. The estimated mean particle diameter was about 60 nm for the core-shell Fe_3_O_4_@SiO_2_ NPs. FE-SEM images obtained from three independently synthesized batches (Figure S4) revealed highly consistent granular morphologies, with particle sizes distributed within a narrow range across all syntheses, confirming both the reproducibility of the process and the size uniformity of the Fe₃O₄@SiO₂ NPs. Figure 3C shows the FE-SEM image of IMA-MMIP@CS, which confirms the formation of the outer CS coating. Figure 3D presents the TEM image of IMA-MMIP@CS, highlighting their distinct multi-layered structure. The heterogeneous composition, consisting of the MMIP followed by the CS layer, is clearly visible due to the contrast in densities and their unique visual characteristics. The dark core in the image represents the Fe_3_O_4_ NPs encapsulated by both the SiO_2_ and the MIP shell (observed as a slightly lighter region surrounding the core). This is surrounded by the outermost CS layer, which completes the final coating of the IMA-MMIP.

Figure 3E presents the EDS analysis of IMA-MMIP@CS, revealing the elemental composition of the synthesized material. The peaks for iron (Fe), oxygen (O), carbon (C), and silicon (Si) confirm the successful encapsulation of Fe_3_O_4_ nanoparticles with SiO_2_ and CS. For comparison, the EDS results of IMA-MMIP were also examined, and these results are provided in the supplementary information (Figure S5). Additionally, the atomic percentages before and after CS coating are detailed in the supplementary information (Table S1). The carbon content increased from 16.66 at% in IMA-MMIP to 20.32 at% in IMA-MMIP@CS, consistent with the introduction of the carbon-rich CS shell. This increase, combined with the presence of N–H and C–H absorption bands in the FT-IR spectrum (Fig. 2A), confirms the successful deposition of chitosan. Peaks for gold (Au) appear due to the gold sputter coating used during the FE-SEM analysis.

### Vibrating sample magnetometer (VSM) analysis

**Fig. 4:**
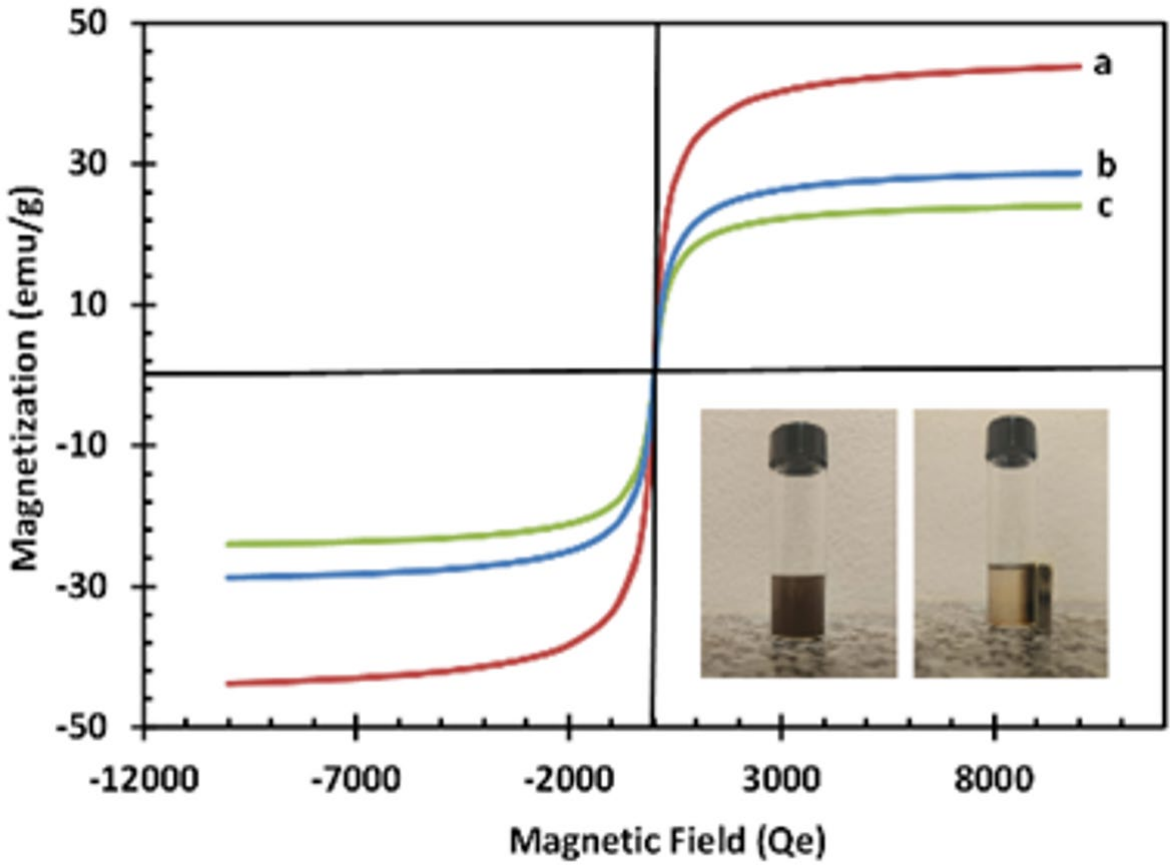
VSM curve of **a**) Fe_3_O_4_, **b**) Fe_3_O_4_@SiO_2_ and **c**) IMA-MMIP@CS.

The magnetic properties of Fe_3_O_4_ NPs, Fe_3_O_4_@SiO_2_ NPs, and IMA-MMIP@CS were characterized by VSM, as illustrated in Fig. 4. The Fe_3_O_4_ NPs exhibited a saturation magnetization of 43.8 emu g^− 1^ at a field of 1.0 × 10^4^ Oe. In comparison, the saturation magnetizations of IMA-MMIPs and IMA-MMP@CS were reduced to at 28.5 and 23.94 emu g^− 1^, respectively. These reductions highlight the significant impact of the progressively thicker modified layers on the surface of Fe_3_O_4_ NPs on their magnetic performance. This confirms the successful coating of SiO_2_, MIP and CS layer on Fe_3_O_4_ NPs, while also demonstrating that the particles retain efficient magnetic properties^62,63^. Although the saturation magnetization decreased from 43.8 emu g^− 1^ for Fe_3_O_4_ to 23.94 emu g^− 1^ after successive coatings, the value remains within the range commonly reported for magnetic nano-systems used in biomedical targeting and separation. Previous studies have shown that saturation magnetization values of 10–30 emu g^− 1^ are sufficient for effective magnetic manipulation under typical biomedical field strengths (0.4–0.8 T)^64–67^. Thus, the proposed system retains adequate magnetic responsiveness for potential in vivo applications under clinically relevant conditions.

### Dynamic light scattering (DLS) and zeta potential analysis

**Fig. 5:**
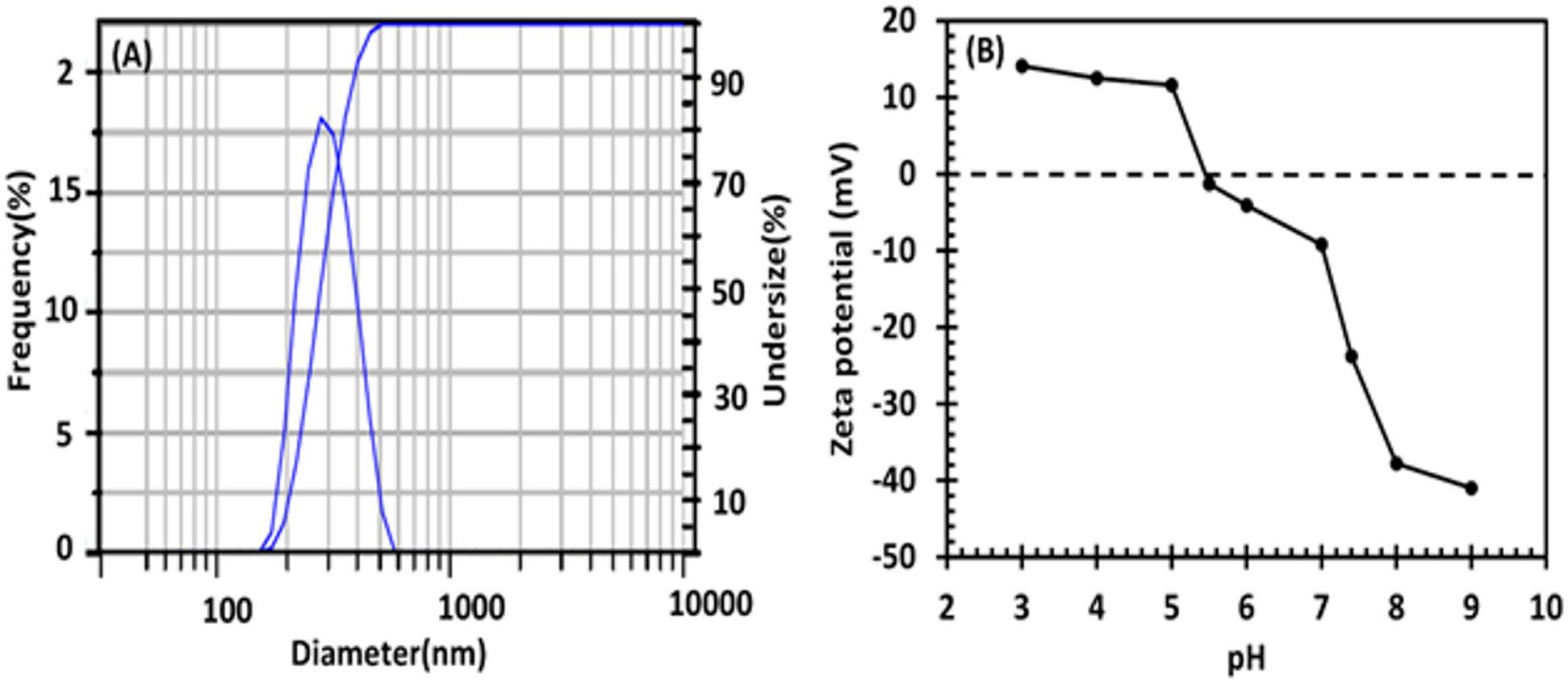
Dynamic light scattering (**A**) and zeta potentials of IMA-MMIP@CS at different pH levels (**B**).

The effective size range for drug delivery using a nanocarrier system is up to 400 nm, allowing for the enhanced permeability and retention (EPR) effect^68,69^. DLS was used to measure the size distribution of IMA-MMIP@CS (Fig. 5A) yielding an average hydrodynamic particle size of approximately 287.7 ± 70.1 nm, indicating a moderately uniform size distribution suitable for drug delivery applications.

Moreover, to assess the stability and surface charge of the fabricated IMA-MMIP@CS particles, the zeta potential was measured in PBS medium across various pH levels, as shown in Fig. 5B. The zeta potential of the IMA-MMIP@CS varied from + 14 to -40 mV over a pH range of 3–9. The isoelectric point (IEP) of IMA-MMIP@CS, where the particles exhibit a neutral surface charge and a zeta potential of zero^68^, was approximately 5.5. This IEP could be due to the presence of amino (–NH_2_) group from CS and partially influence of the functional monomers used in MIP layer^70^. As the pH increased, the protonation degree of the –NH_2_ groups in IMA-MMIP@CS decreased, causing a gradual shift of the zeta potentials from positive to negative. This implies that the concentration of protonated –NH_2_ groups (-NH^3+^) is directly proportional to the zeta potentials of the nanocarrier. A charge conversion was also observed between pH 5.0 and 6.0, suggesting that IMA-MMIP@CS is unstable within this pH range. The instability could be beneficial in terms of releasing the drug^71^. The physical stability of the IMA-MMIP@CS suspension was evaluated via visual inspection and photographic documentation over 7 days at room temperature (Figure S6). No visible precipitation, turbidity changes, or phase separation were observed. Furthermore, the surface of IMA-MMIP@CS was negatively charged at pH 5.5–9.0. This negative charge effectively protected the nanocarrier from non-specific uptake by the reticuloendothelial system and promoted targeted release^72,73^. These results suggest that the IMA-MMIP@CS system is well suited for enhancing drug release in acidic environments, such as those found in tumors and inflamed tissues.

### The template/functional monomer ratio optimization

The appropriate ratio of functional monomers to template molecules is critical in creating highly specific molecular recognition sites. An optimal ratio ensures strong and selective interactions with the template, thereby enhancing the performance of the imprinted polymer. However, an excess of functional monomers during polymerization can lead to non-specific binding, compromising the selectivity of the polymer^72^. Thus, choosing the optimal functional monomer/template ratio plays an important role for achieving effective molecular imprinting.

To optimize the recognition sites for IMA, five MMIPs (1–5) and the corresponding MNIPs were prepared using various ratios of APTES (functional monomer) and IMA (template), with a constant APTES concentration and varying IMA concentrations from 0.0 to 0.05 mmol. The DLC and IF values of the fabricated MMIPs were evaluated (Eqs. 1 and 3, Table 2). Among them, MMIP-3, with an APTES/IMA ratio of 5:1, exhibited the highest DLC and IF values, while the corresponding MNIP showed the lowest levels, indicating that this ratio provided the most effective recognition sites. All measurements were performed in triplicate, and DLC values are reported as mean ± SD. Statistical analysis (one-way ANOVA) confirmed that the 5:1 ratio (654 ± 10 mg/g) was significantly higher (*p* < 0.05) than other ratios. Thus, MMIP-3 was selected as the IMA nanocarrier for subsequent studies.

**Table 2:** Effect of the ratio of template to functional monomers on the Loading capacities of five IMA-MMIPs (mean ± SD, *n* = 3).

MMIPs	IMA (mmol)	APTES (mmol)	TEOS (mmol)	Fe_2_O_3_@SiO_2_ (mg)	DLC (mg/g)	IF
1	0.050	0.100	0.200	20.00	542.11 ± 12	4.54
2	0.033	0.100	0.200	20.00	598.41 ± 15	5.01
3	0.020	0.100	0.200	20.00	654.08 ± 10	5.48
4	0.014	0.100	0.200	20.00	420.14 ± 11	3.52
5	0.010	0.100	0.200	20.00	387.98 ± 14	3.25
MNIP	0.000	0.100	0.200	20.00	119.24 ± 9	-

### Drug loading capacity and selectivity

**Fig. 6:**
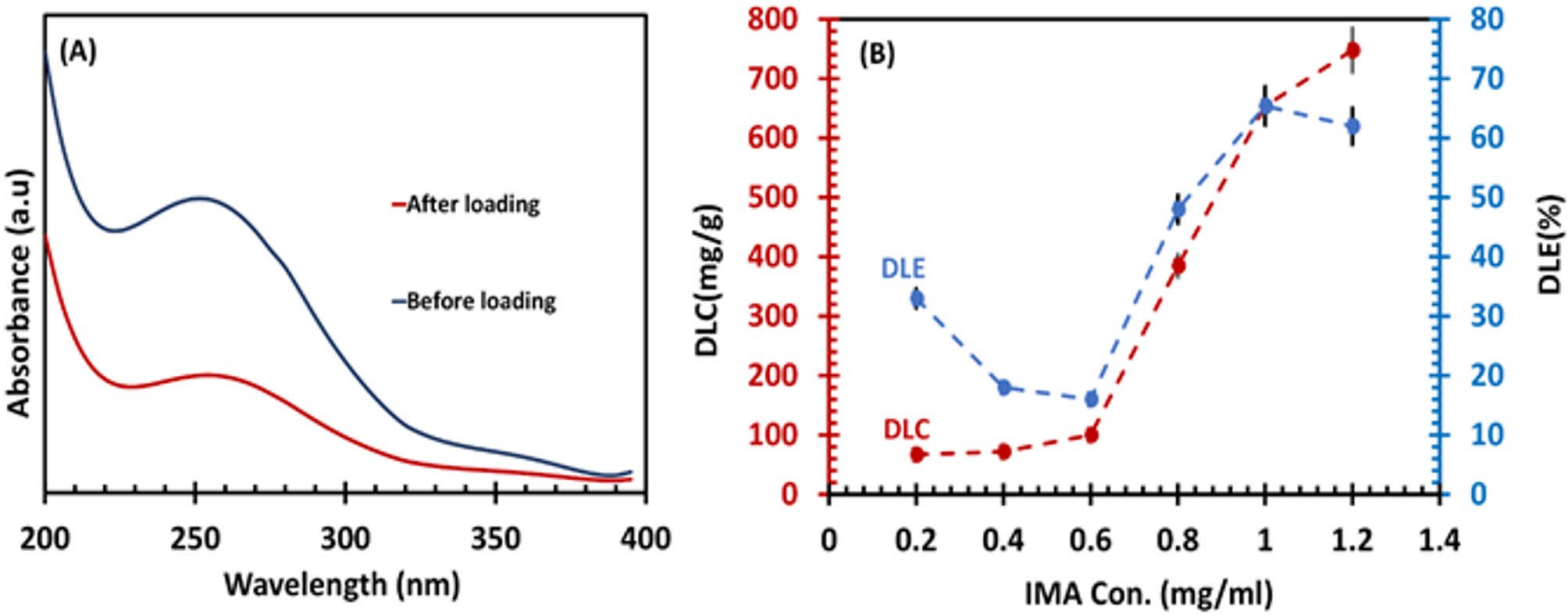
(**A**)UV-Vis spectrum of IMA (0.05 mg/ml) before (blue one) and after (red one) loading on MMIPs. (**B**) DLC and DLE curves for IMA loading on MMIPs.

To investigate the capacity of the proposed MMIP nanocarrier for IMA loading, UV-Vis spectrophotometer was used. The UV-Vis spectra (Fig. 6A of free IMA (0.05 mg/ml) showed a maximum absorbance at 255 nm (blue curve), while after loading onto the nanocarrier, the absorbance of IMA decreased (red curve), confirming the successful loading of drugs onto the nanocarrier.

To achieve the highest drug loading capacity, various concentrations of IMA (0.2, 0.4, 0.6, 0.8, 1.0 and 1.2 mg/ml) were mixed with a constant amount of nanocarrier (10 mg). Then the DLC and DLE were evaluated using UV-Vis spectra and calibration curves. Upon analysis, it was discovered that the MMIP had a maximum capacity of 654 ± 10 mg/g, with more than 65.4% of IMA loaded at concentration of 1.0 mg/ml (Fig. 6B).

The specificity and selectivity of MMIP towards IMA were tested in the presence of structurally similar compounds, including Sorafenib and Regorafenib. As shown in Table 3, the DLC and IF values of MMIP for IMA are several times higher than those of Sorafenib and Regorafenib, indicating a higher binding affinity and specificity of MMIP for IMA, with minimal interference from other substances. In addition, the comparison of IF values between MMIP and MNIP further confirms the stronger binding specificity of MMIP.

**Table 3:** Selectivity of MMIP for similar structures (mean ± SD, *n* = 3).

Drug	DLC (mg/g)		IF
	MMIP	MNIP	
Imatinib	654.08 ± 10	119.24 ± 9	5.48
Sorafenib	97.66 ± 7	84.79 ± 11	1.15
Regorafenib	89.85 ± 9	80.31 ± 8	1.11

### Releasing behavior of IMA from IMA-MMIP and IMA-MMIP@CS

In vitro release studies were conducted on IMA-MMIP and IMA-MMIP@CS samples at two pH levels (5.5 and 7.4) at 37 °C. These pH values were chosen because blood typically has a pH of 7.4, while the extracellular environment of tumor tissues is more acidic, ranging from 6.5 to 7.4, and upon reaching endosomes and lysosomes, the pH often decreases further to values between 5.0 and 5.5^74^. At the start of the release process, IMA molecules are released from both carriers at a rapid rate. Over time, however, the release slows down significantly. The initial burst is due to the strong release of IMA molecules that were adsorbed onto the surface of imprinted site. As the release progresses, the diffusion rate decreases, becoming much slower. This slower phase is attributed to the IMA molecules trapped within the deeper imprinted sites of the MMIP and the polymeric matrix of CS, which take longer to release.

**Fig. 7:**
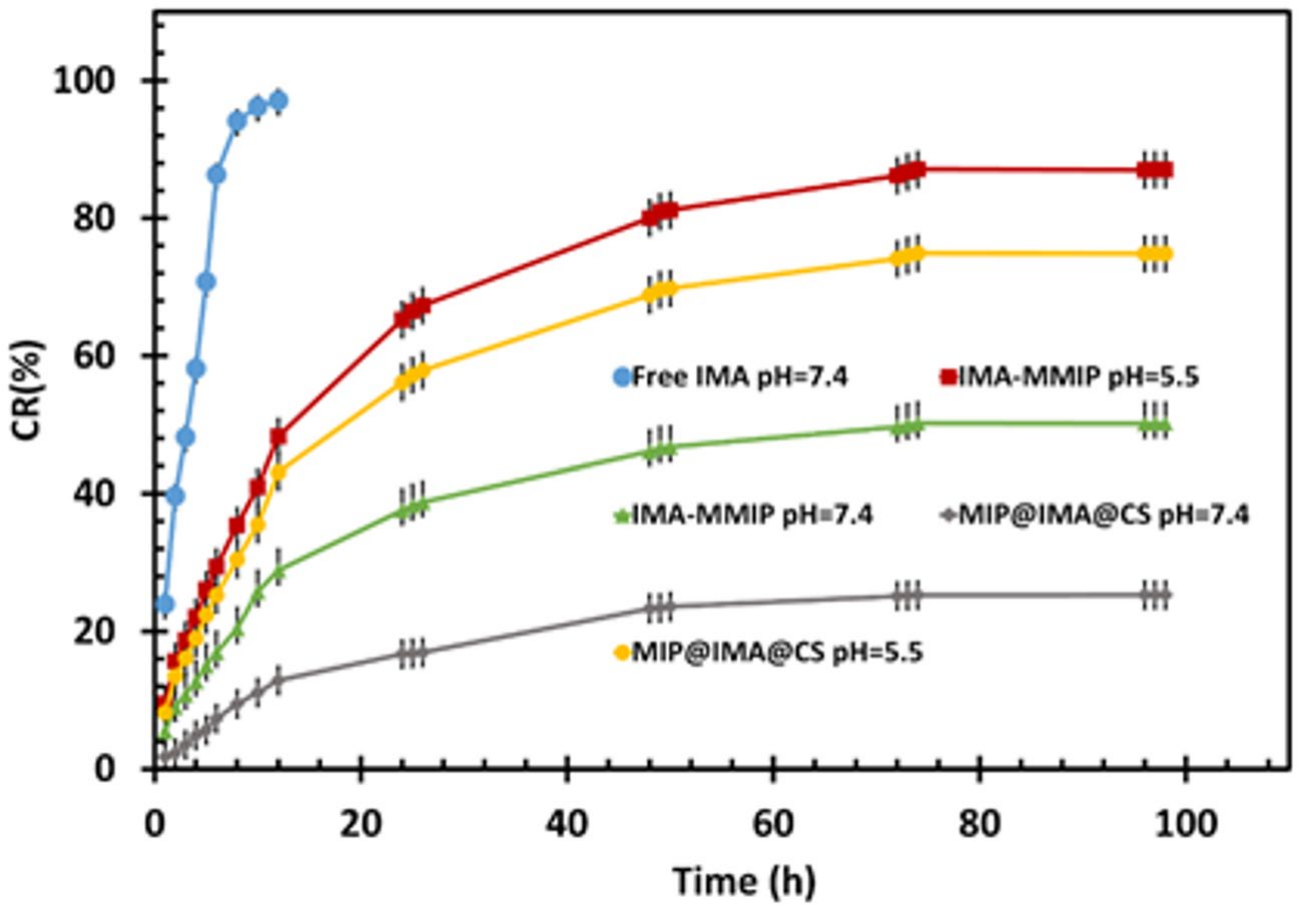
Cumulative release profiles of free IMA in PBS (pH 7.4) and IMA from IMA-MMIP and IMA-MMIP@CS nanocarriers at pH 5.5 and 7.4 at 37 °C over a 96 h period. Data are presented as mean ± standard deviation (*n* = 3).

Figure 7 illustrates the IMA release profiles for IMA-MMIP and IMA-MMIP@CS at two different pH levels. Although the overall trends of the curves are similar, drug release is significantly higher at the acidic pH of 5.5 (87.03%) compared to the neutral pH of 7.4 (50.13%). This behaviour can be attributed to the protonation of free amine groups present in both IMA and the APTES monomers forming the MMIP shell under acidic conditions. Protonation alters the chemical environment of the imprinted binding sites and weakens the hydrogen-bonding interactions between MMIP and IMA, thereby facilitating drug release. In addition, the increased density of positively charged –NH₃⁺ groups generates electrostatic repulsion within the MMIP network, leading to polymer swelling and partial deformation of the imprinted cavities. The combined effects of weakened intermolecular interactions and polymer expansion reduce the binding affinity of IMA, promoting its diffusion and release under acidic conditions^75,76^. Additionally, the relatively low cumulative release at pH 7.4 is beneficial, as it suggests that IMA-MMIP can remain stable for a longer duration in normal tissues, reducing potential adverse effects^77,78^.

The IMA release profile of IMA-MMIP@CS demonstrates a significantly slower release compared to IMA-MMIP. This behaviour reflects the controlled release of IMA from IMA-MMIP@CS under the tested pH conditions. After 72 h, only 25% of IMA was released at pH 7.4, whereas 74.5% was released at pH.

5.5. This difference highlights the role of the CS layer in IMA-MMIP@CS. The CS layer acts as a protective shield, selectively swelling in acidic environments and contracting at higher pH levels. This behavior is attributed to the pH-dependent charge properties and charge density of IMA-MMIP@CS, governed by the –NH_2_ groups on the surface of the CS^24^. This functionality ensures controlled release in the two pH ranges^77^. Consequently, the CS shell functions as a smart barrier, minimizing IMA release at pH 7.4 while enabling substantial drug release under acidic conditions that mimic the tumor microenvironment. Interestingly, the cumulative release of IMA from IMA-MMIP@CS at pH 5.5 (74.5%) was slightly lower than that of the uncoated IMA-MMIP (87.0%). This reduction can be explained by incomplete swelling and partial entrapment of drug molecules within the cross-linked CS network, which introduces an additional diffusional barrier. Such incomplete release is a well-documented phenomenon in chitosan-based and other polymer-coated drug delivery systems, where the coating’s thickness, crosslinking density, and degree of swelling directly influence the rate and extent of drug release. This behavior represents a common trade-off^79,80^ in controlled-release systems: the CS coating slightly reduces the overall release yield but provides superior control and stability under physiological conditions, thereby reducing premature release and improving selectivity for tumor-like environments. Table S4 provides quantitative comparisons with previously reported targeted drug delivery systems for IMa and similar TKIs (dasatinib, nilotinib). As indicated, most of the reported carrier systems for IMA and related drugs are based on nanoparticles modified with CS or CS-containing hybrid materials. While these systems demonstrate varying degrees of drug loading, encapsulation efficiency, and release kinetics, many exhibit relatively short release durations or limited pH-responsiveness. In contrast, the IMA-MMIP@CS developed in this work combines molecular imprinting with surface modification, achieving high drug encapsulation and extended, pH-responsive release over 96 h. This design addresses the limitations of prior systems by providing tailored binding sites for selective drug recognition, enhanced stability, and controlled release under acidic conditions, thereby improving potential therapeutic efficacy^39,81–84^.

### Drug release kinetics and mechanisms

To elucidate the IMA release mechanisms from IMA-MMIP and IMA-MMIP@CS, drug release kinetics were analyzed using Zero-order, First-order, Higuchi, Korsmeyer-Peppas, and Hixson-Crowell models. Zero-order kinetics describes a constant release rate independent of remaining drug quantity, often used in controlled-release systems^85^. First-order kinetics involves a release rate proportional to the drug’s concentration gradient, common in water-soluble drugs in porous matrices^86^. The Higuchi model applies to matrix systems, assuming constant diffusivity and significant initial drug concentration^87^. The Korsmeyer-Peppas model characterizes Fickian (diffusion-driven) and non-Fickian (polymer relaxation and swelling) mechanisms^88,89^. The Hixson-Crowell model explains release via surface area changes during dissolution, typical in tablet formulations^90^.

**Fig. 8:**
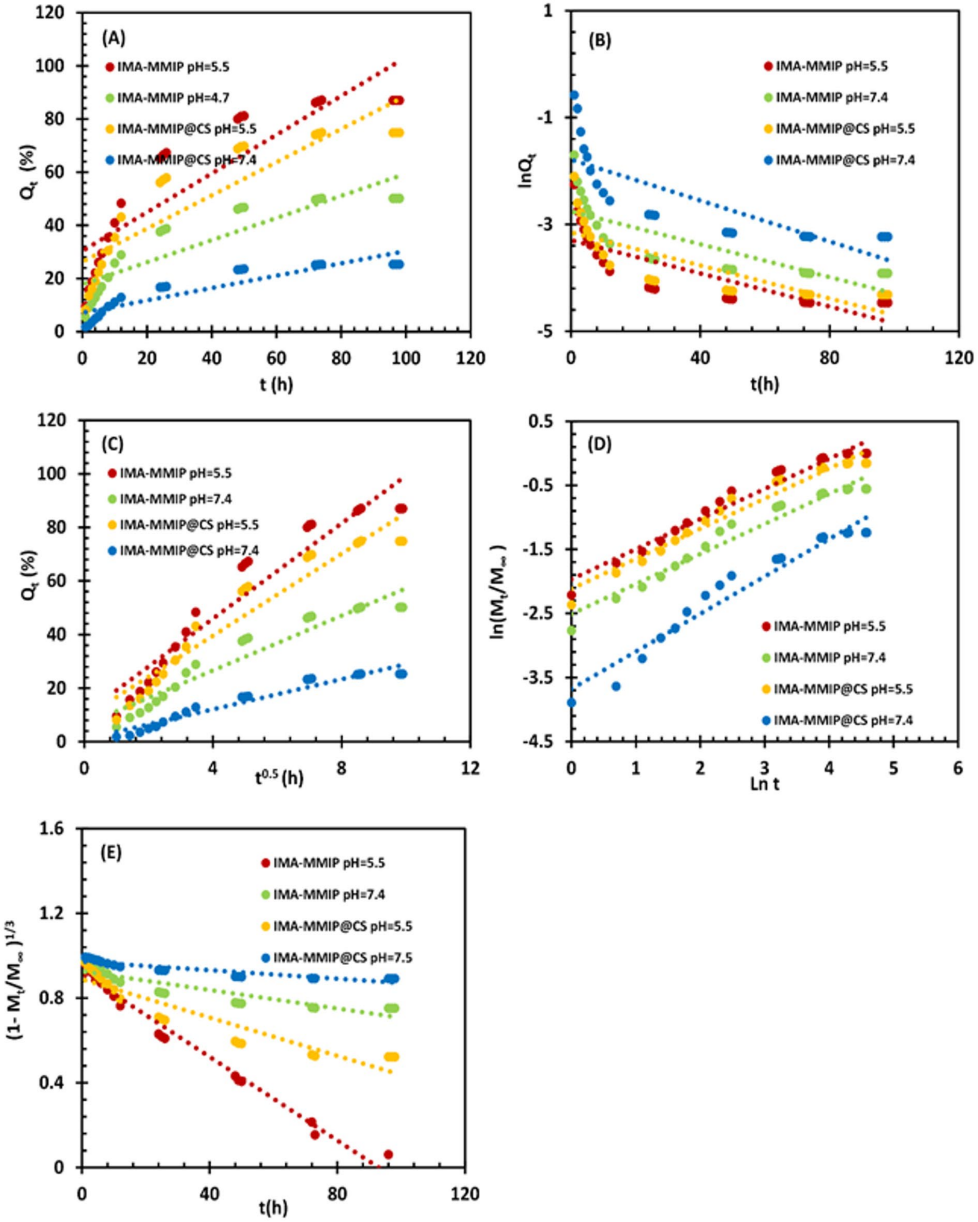
Graphs of the experimental adjustment to the (**A**) Zero-order, (**B**) First-order, (**C**) Higuchi, (**D**) Korsmeyer-Peppas, and (**E**) Hixson-Crowell model for IMA release from IMA-MMIIP@CS.

Figure 8 presents the experimental data fitted to Zero-order (Fig. 8A), First-order (Fig. 8B), Higuchi (Fig. 8C), Korsmeyer-Peppas (Fig. 8D), and Hixson-Crowell (Fig. 8E) models at different pH levels, with the corresponding parameters for each model summarized in Table 4. Among the evaluated kinetic models, the Korsmeyer–Peppas and Higuchi models exhibited the highest correlation coefficients (R²), indicating the best fit for the drug release profiles of both IMA-MMIP and IMA-MMIP@CS at different pH levels. In contrast, the zero-order, first-order, and Hixson–Crowell models showed relatively lower R² values, particularly in the case of IMA-MMIP@CS. These results suggest that drug release from the systems does not follow a constant rate (zero-order), nor is it purely concentration-dependent (first-order), nor governed predominantly by changes in surface area and volume (Hixson–Crowell). However, Hixson–Crowell only had a slightly better fit at IMA-MMIP pH 5.5, implying some role of erosion or particle surface area reduction at this pH. Instead, the release appears to be regulated by a combination of diffusion and polymer relaxation mechanisms, as described by the Korsmeyer–Peppas and Higuchi models. The strong correlation with the Higuchi model points to diffusion as a key factor in the IMA release process. Given the limitations of the Higuchi model in accurately describing drug release from polymer-based systems, the release data were also analyzed using the exponential Korsmeyer–Peppas equation, a widely applied model for characterizing release profiles from polymeric matrices. In this approach, the release exponent (n) provides insight into the underlying release mechanism. When *n* ≤ 0.45 (Case I), the process is dominated by Fickian diffusion. Values of 0.45 < *n* < 0.89 correspond to non-Fickian (anomalous) transport, where both diffusion and polymer matrix relaxation contribute to drug release. An exponent of *n* = 1 represents zero-order kinetics, in which release is primarily driven by polymer relaxation or matrix erosion. Finally, *n* > 0.89 (Case II) suggests that polymer relaxation is the predominant mechanism controlling the release process. For the prepared delivery systems, the estimated n values ranged from 0.46 to 0.58, which is greater than 0.45, indicating non-Fickian (Anomalous) release. IMA release from the IMA-MMIP occurs via both diffusion and polymer erosion. A comparative analysis of IMA-MMIP and IMA-MMIP@CS revealed that the release kinetics vary slightly between pH 7.4 and pH 5.5. In general, the rate constants (k values) are higher at pH 5.5 for most models, indicating a faster release of the drug under acidic conditions. This suggests that the systems are sensitive to pH, with acidic environments promoting quicker drug release, potentially due to increased swelling or erosion of the polymer. Furthermore, IMA-MMIP@CS typically exhibits lower rate constant values (k), confirming a more controlled and diffusion-dominated release compared to IMA-MMIP. This observation highlights the role of the CS coating in regulating drug release, likely by enhancing matrix stability and slowing down the erosion process, which leads to a more sustained release profile in neutral or basic conditions.

Under acidic conditions, protonation of the chitosan amino groups induces electrostatic repulsion within the polymer network, causing the CS layer to swell. This facilitates water penetration into the carrier and weakens hydrogen bonding and electrostatic interactions between imatinib and the imprinted binding sites within the MMIP matrix. Consequently, drug release is enhanced at lower pH. This behavior, combining diffusion and polymer relaxation, aligns with the anomalous (non-Fickian) transport observed in the Korsmeyer–Peppas model.

**Table 4:** Release kinetic parameters of IMA.

Model	Equation	Parameter	IMA-MMIP		IMA-MMIP@CS	
			pH = 7.4	pH = 5.5	pH = 7.4	pH = 5.5
Zero-order	$\:{\mathbf{Q}}_{\mathbf{t}}-{\mathbf{Q}}_{0}={\mathbf{k}}_{0}.\mathbf{t}$	K_o_	0.4163	0.7287	0.2322	0.6247
		R^2^	0.6345	0.6247	0.6307	0.6210
First-order	$\:{\mathbf{Q}}_{\mathbf{t}}-{\mathbf{Q}}_{0}={\mathbf{e}}^{-{\mathbf{k}}_{1}.\mathbf{t}}$	K_1_	0.0154	0.0155	0.0192	0.0155
		R^2^	0.7846	0.791	0.8219	0.7881
Higuchi	$\:{\mathbf{Q}}_{\mathbf{t}}={\mathbf{k}}_{\mathbf{H}}{.\mathbf{t}}^{0.5}$	K_H_	5.0969	8.9062	2.8115	7.6408
		R^2^	0.9184	0.9229	0.9408	0.9208
Korsmeyer _ Peppas	$\:{\mathbf{M}}_{\mathbf{t}}/{\mathbf{M}}_{{\infty\:}}={\mathbf{k}}_{\mathbf{K}\mathbf{P}}{.\mathbf{t}}^{\mathbf{n}}$	K_K−P_	0.1203	0.1398	0.0253	0.0812
		n	0.4703	0.4708	0.5845	0.4691
		R^2^	0.9584	0.9605	0.9497	0.9553
Hixson-Crowell	$\:{(1-{\mathbf{M}}_{\mathbf{t}}/{\mathbf{M}}_{{\infty\:}})}^{1/3}={\mathbf{k}}_{\mathbf{H}\mathbf{C}}.\mathbf{t}$	K_H−C_	0.0022	0.0099	0.0010	0.0045
		R^2^	0.8129	0.9810	0.8268	0.8651

### In vitro cytotoxicity

**Fig. 9:**
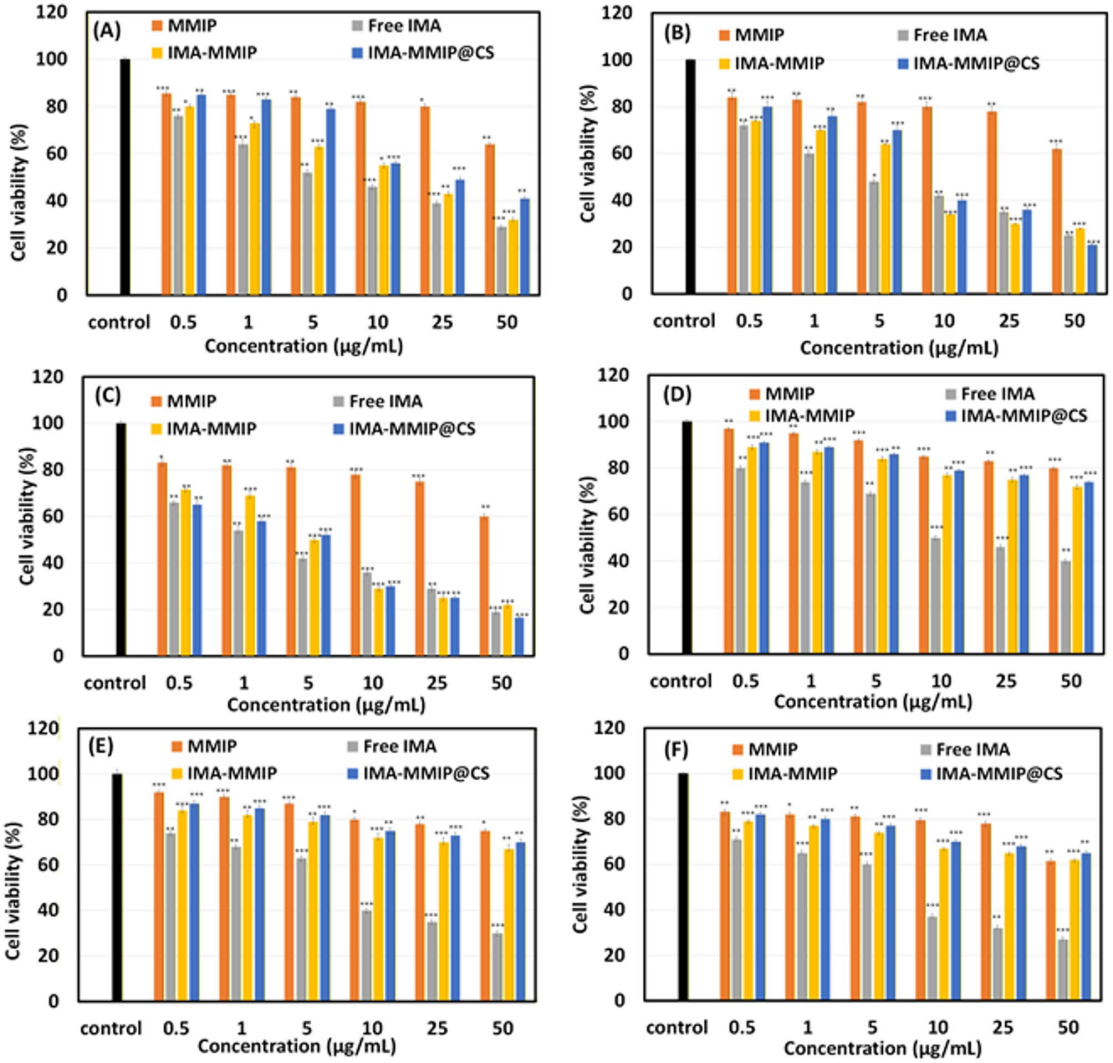
In vitro cytotoxicity of free IMA, MMIP, IMA-MMIP, and IMA-MMIP@CS evaluated by MTT assay in K562 cells (**A**–**C**) and PBMCs (**D**–**F**) after 24 h (**A**, **D**), 48 h (**B**, **E**), and 72 h (**C**, **F**) of incubation. Cell viability is expressed as a percentage relative to untreated control cells. Data are presented as mean ± SD (*n* = 3). Statistical significance was determined relative to the control group (*p* < 0.05 (*),*p < 0.01*(**), *p* **<** 0.001(***)).

To evaluate the cytotoxicity of the developed carriers compared to free IMA, the MTT assay was performed using K562 leukemia cells (Fig. 9A-C) and PBMCs Cells (Fig. 9D-F) were treated for 24, 48, and 72 h, with drug concentrations kept consistent across all drug-containing samples.

The MMIP alone exhibited minimal cytotoxicity toward both cell lines, even at higher concentrations and longer incubation times. Over 60% cell viability was maintained following treatment with 50 µg/mL of MMIP for 72 h, confirming its good biocompatibility and suitability as a safe drug delivery system. In contrast, free IMA induced pronounced cytotoxic effects in both K562 cells and PBMCs, with approximately 60% cell death observed after exposure to 50 µg/mL for 24 h. Interestingly, at higher concentrations and after 48 h of incubation, delivery of IMA via IMA-MMIP and IMA-MMIP@CS resulted in more effective growth inhibition in K562 cells compared to the free drug (Fig. 9B). After 72 h, IMA-MMIP@CS (Fig. 9C) exhibited higher cancer cell inhibition than both free IMA and IMA-MMIP, highlighting the sustained, time-dependent release conferred by the CS coating. Importantly, PBMCs retained significantly higher viability across all tested concentrations of IMA-MMIP and IMA-MMIP@CS compared to K562 cells, indicating that the nanocarriers induced substantially lower cytotoxicity in normal PBMCs than free IMA, even at higher concentrations and longer incubation times (Fig. 9D-F). These results demonstrate that encapsulation of IMA within MMIP nanoparticles, particularly with CS surface modification, reduces nonspecific toxicity toward normal cells while enhancing selective, time-dependent anticancer activity. The IC₅₀ values were approximately 2.5, 5.4, 7.2, and 91.7 µg/mL for free IMA, IMA-MMIP, IMA-MMIP@CS, and MMIP, respectively. Although free IMA exhibited the lowest IC₅₀ due to its immediate availability and rapid cellular uptake, the higher IC₅₀ values observed for IMA-MMIP and IMA-MMIP@CS at a fixed time point reflect their sustained, time-dependent drug release behavior. Notably, IMA-MMIP@CS demonstrated enhanced long-term cytotoxicity against K562 cells while markedly reducing nonspecific toxicity toward PBMCs, indicating that the primary advantage of these nanocarriers lies in improved therapeutic selectivity and controlled release rather than maximal immediate potency.

## Conclusions

In summary, this study successfully developed a novel pH-responsive drug delivery system by using a CS biopolymer shell to cap the drug-loaded MMIP. This design combines the dual stimuli-responsive nature of the polymer with magnetic properties to achieve precise drug release in the acidic environment typical of tumor tissues. Using IMA as a model anticancer drug, the release behavior from both IMA-MMIP and IMA-MMIP@CS was examined under different pH conditions. The results demonstrated that the IMA MMIP@CS system significantly outperformed the uncoated MMIP system, achieving controlled, pH-responsive drug release tailored to simulate the acidic conditions of tumor environments (pH 5.5). At pH 5.5, cumulative IMA release from IMA-MMIP@CS reached 74.5%, compared with only 21% at pH 7.4, indicating an approximately 3.5-fold higher release under acidic versus neutral conditions. The drug release was primarily governed by expansion of the CS shell and weakening of hydrogen bonds as the pH shifted to a more acidic state. Kinetic analysis showed that the release mechanism was largely driven by diffusion, as described by the Korsmeyer–Peppas model.

Furthermore, in vitro cytotoxicity studies demonstrated that both IMA-MMIP and IMA-MMIP@CS exhibited enhanced anticancer activity against K562 cells while maintaining substantially higher viability in normal PBMCs compared to free IMA. The IMA-MMIP@CS, in particular, showed time-dependent cytotoxicity, consistent with its sustained, pH-responsive release behaviour. These findings highlight the potential of IMA-MMIP@CS as a biocompatible, targeted, and efficient drug delivery platform for tumor-specific therapy.

## Supplementary Information

Below is the link to the electronic supplementary material.

Supplementary Material 1

## Data Availability

The data that support the findings of this study are available from the corresponding author upon reasonable request.
