# Supplementary material for: Magnetic molecularly imprinted polymer coated with chitosan shell for enhanced controlled drug release
Source: Sci Rep. 2026 Feb 25;16:11015. doi: 10.1038/s41598-026-41273-0 (PMC13043948; doi:10.1038/s41598-026-41273-0)
Supplement: Supplementary file 1 — Supplementary Material 1 [file 41598_2026_41273_MOESM1_ESM.docx]

**Supporting information**

Magnetic Molecularly Imprinted Polymer Coated with Chitosan Shell for Enhanced Controlled Drug Release

Najva Sadri^1^, Mohammad Mazloum-Ardakani^1*^, Yvonne Joseph^2^, Parvaneh Rahimi^2*^

^1^Department of Chemistry, Faculty of Science, Yazd University, Yazd 89195-741, Islamic Republic of Iran; najva.sadri@stu.yazd.ac.ir;

^2^Institute of Nanoscale and Biobased Materials, Faculty of Materials Science and Technology, Technische Universität Bergakademie Freiberg, 09599 Freiberg, Germany. [yvonne.joseph@esm.tu-freiberg.de](mailto:yvonne.joseph@esm.tu-freiberg.de)

*Corresponding authors: E-mail: [mazloum@yazd.ac.ir](mailto:mazloum@yazd.ac.ir) (M. Mazloum-Ardakani), [Parvaneh.Rahimi@esm.tu-freiberg.de](mailto:Parvaneh.Rahimi@esm.tu-freiberg.de) (P. Rahimi)

The concentration of imatinib was quantified using UV–Vis spectroscopy at a wavelength of 255 nm. Calibration curves were established in deionized water, PBS (pH 7.4), and PBS (pH 5.5), as shown in Figure S1. Drug loading, release, and template leaching experiments were calculated based on these calibration curves.


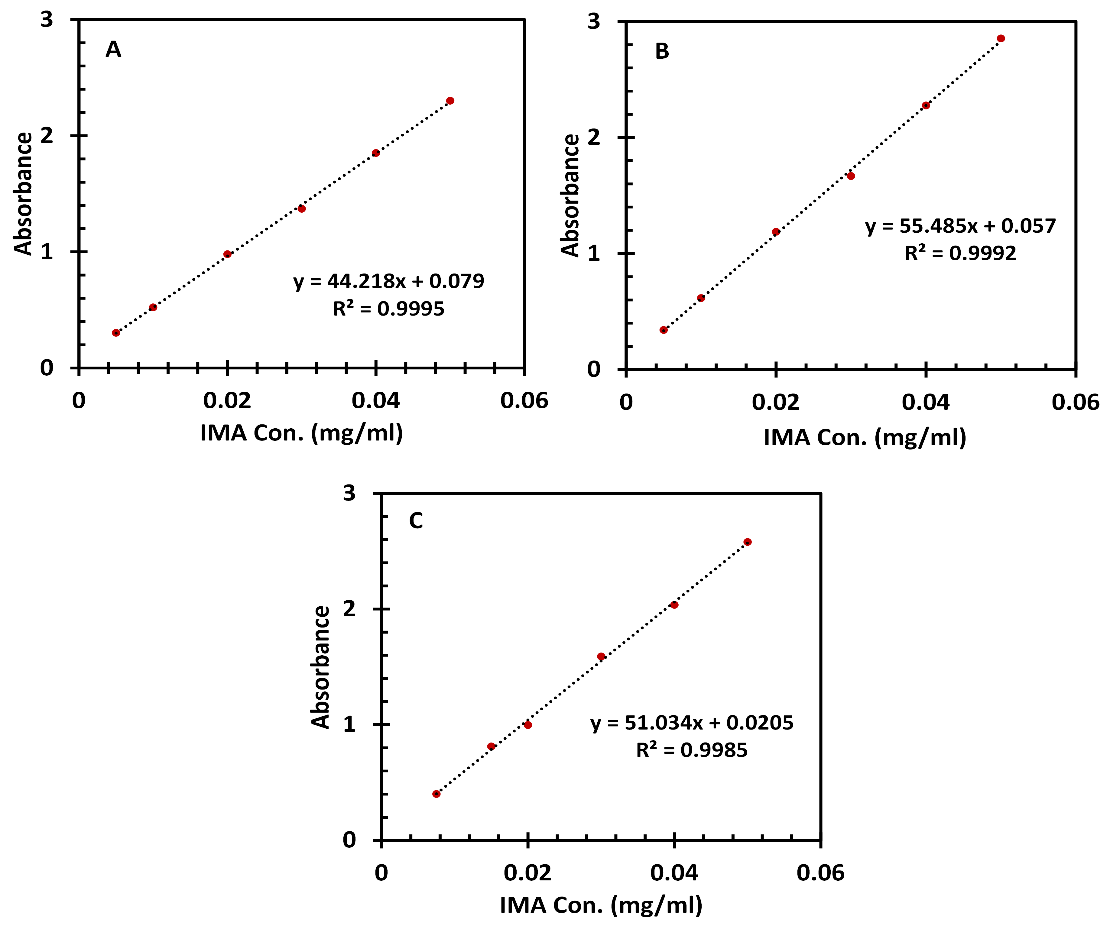


Figure S1. Calibration curve of IMA at (A) deionized water, (B) PBS pH 7.4 and (C) PBS pH 5.5


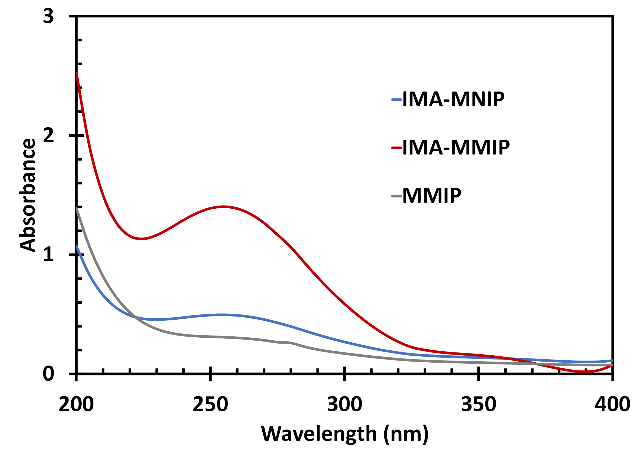


Figure S2. UV–vis spectrum of MMIP, drug loaded MNIP (IMA-MNIP) and drug loaded MMIP (IMA-MMIP)


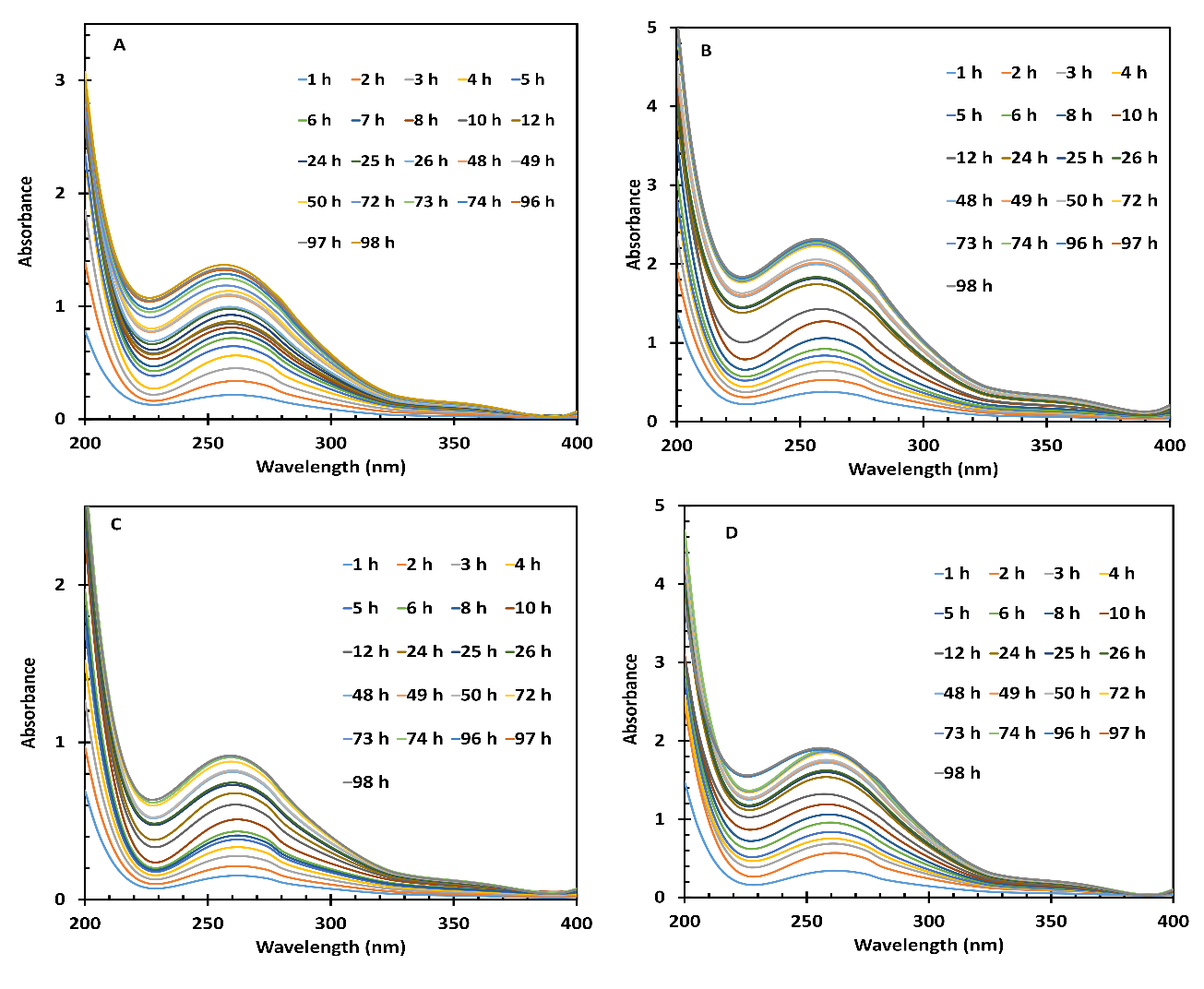


Figure S3. UV-Vis spectrum of releasing drug from (A)IMA-MMIP at pH 7.4, (B) IMA-MMIP at pH 5.5, (C) IMA-MMIP@CS at pH 7.4 and (D) IMA-MMIP@CS at pH 5.5


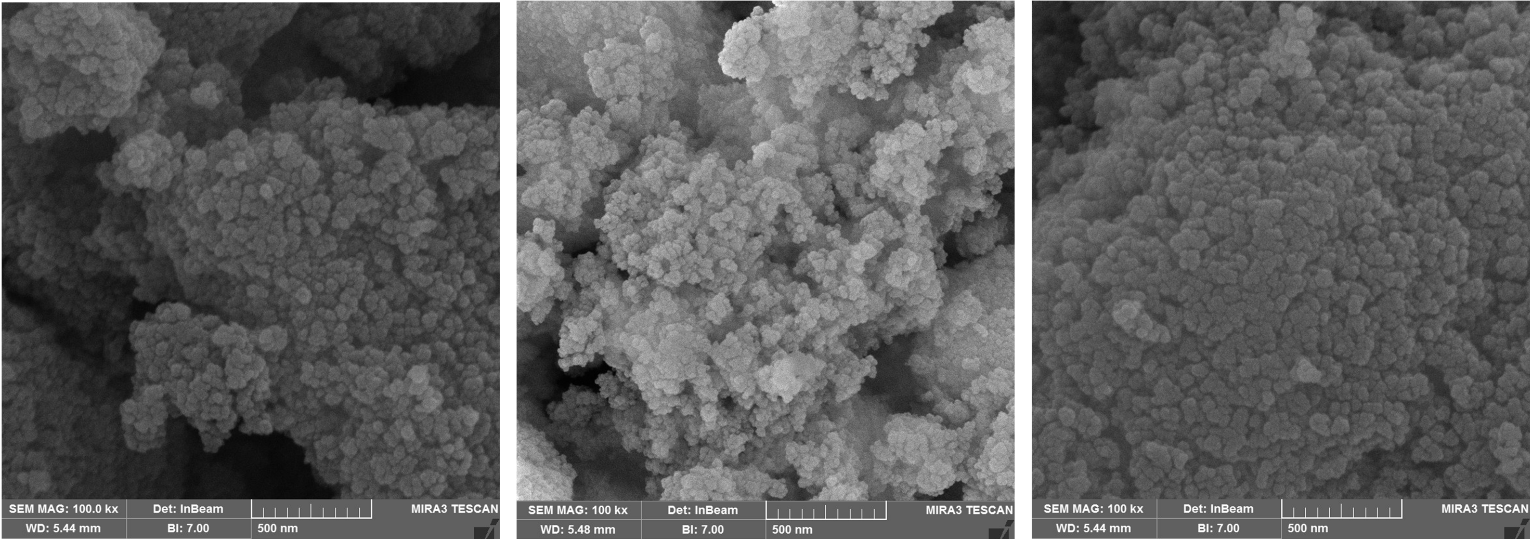


Figure S4. FE- SEM images of Fe₃O₄@SiO₂ from three independent syntheses under identical stirring conditions, showing reproducible morphology and particle sizes within a narrow range.


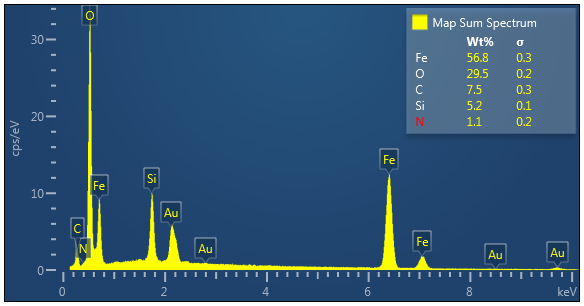


Figure S5. EDS spectrum of IMA-MMIP.


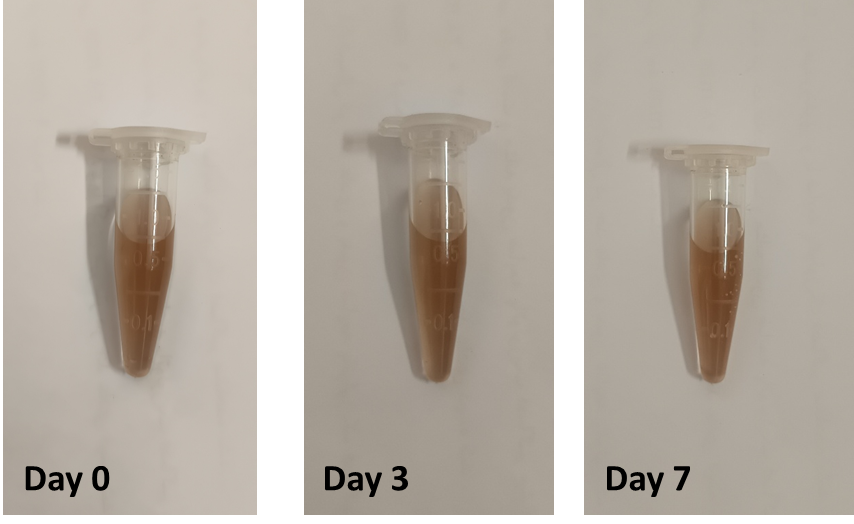


Figure S6. Photographic images of the magnetic molecularly imprinted polymer (IMA-MMIP@CS) dispersion in phosphate-buffered saline (PBS, pH 7.4) taken at **Day 0, Day 3, and Day 7**

| Element | Atomic % (Before CS Layer) | Atomic % (After CS Layer) |
| --- | --- | --- |
| C | 16.66 | 20.32 |
| O | 49.28 | 45.63 |
| Si | 4.90 | 3.60 |
| Fe | 27.13 | 26.60 |

Table S1. EDS Data Comparison

Table S2. Raw drug loading calculation data for IMA-MMIP@CS

| Sample | Initial concentration C₀ (mg/mL) | final concentration C_f_ (mg/L) | Volume (mL) | Mass of carrier (mg) | DLC* (mg/g) |
| --- | --- | --- | --- | --- | --- |
| 1 | 1.0 | 0.346 | 10.0 | 10.0 | 654.0 |

* Drug loading capacity (DLC) was calculated using the equation: DLC (mg/g) = (C₀ − Cf) × V / m, where C₀ and Cf are the initial concentration of IMA and the final drug concentration in the supernatant solution, V is the solution volume, and m is the mass of the carrier.

Table S3. Raw template leaching data of IMA from MMIP during washing

| Washing cycle | Absorbance (255 nm) | Concentration (mg/mL) |
| --- | --- | --- |
| 1 | 0.259 | 0.004 |
| 2 | 0.103 | 0.0005 |
| 3 | 0.086 | 0.0001 |
| 4 | ND* | ND |

*ND: not detectable. Washing was continued until no detectable imatinib was observed in the supernatant, confirming complete template removal.

Table S4. Comparison of drug loading and release for imatinib and related TKIs.

| Drug | Carrier system | DEE* (%) | DLC(%) | Release profile | Reference |
| --- | --- | --- | --- | --- | --- |
| Dasatinib | Lecithin–chitosan hybrid nanoparticles (non-imprinted) |  | 1.53 | Sustained release up to 48 h | [1] |
| Nilotinib | Magnetic graphene oxide core–shell NPs *(CSP, CDSP, CTSP) | --- | 40 | CSP: ~95% up to 24 h  CDSP: ~97.5% up to 37 h  CTSP: ~99% up to 60 h | [2] |
| Imatinib | Fe@TiO₂ / magnetic NPs — pH-sensitive magnetic NPs |  | 40.8 | Release up to 72 h  83% | [3] |
| Imatinib | Chitosan-based polymeric nanoparticles | 68.52 | --- | Cumulative release ≈ 86.45% | [4] |
| Imatinib | Chitosan-modified magnetic nanoparticles | --- | 52 | Release up to 2 h  pH 5.5 ~ 100 % ;  pH 7.4 ~ 40 % | [5] |
| Imatinib | Chitosan coated MMIP | 65 | 65.4 | Release up to 96 h  pH 5.5 =74.5% pH 7.4 = 25% | This work |

*Drug entrapment efficiency (DEE), core-shell particles (CSP), core-dual shell particles (CDSP) and core-triple shell particles (CTSP)

References

[1] A. Mehandole *et al.*, “Dasatinib loaded mucoadhesive lecithin-chitosan hybrid nanoparticles for its augmented oral delivery, in-vitro efficacy and safety,” *Int. J. Pharm.*, vol. 651, p. 123784, Feb. 2024, doi: 10.1016/J.IJPHARM.2024.123784.

[2] M. Zhalechin, S. M. Dehaghi, M. Najafi, and A. Moghimi, “Magnetic polymeric core-shell as a carrier for gradual release in-vitro test drug delivery,” *Heliyon*, vol. 7, no. 5, p. e06652, May 2021, doi: 10.1016/j.heliyon.2021.e06652.

[3] S. Bhullar, N. Goyal, and S. Gupta, “In-vitro pH-responsive release of imatinib from iron-supplement coated anatase TiO2 nanoparticles,” *Sci. Rep.*, vol. 12, no. 1, pp. 1–12, Dec. 2022, doi: 10.1038/S41598-022-08090-7;SUBJMETA.

[4] S. Bhattacharya, “Fabrication and characterization of chitosan-based polymeric nanoparticles of Imatinib for colorectal cancer targeting application,” *Int. J. Biol. Macromol.*, vol. 151, pp. 104–115, May 2020, doi: 10.1016/J.IJBIOMAC.2020.02.151.

[5] Z. Karimi Ghezeli, M. Hekmati, and H. Veisi, “Synthesis of Imatinib-loaded chitosan-modified magnetic nanoparticles as an anti-cancer agent for pH responsive targeted drug delivery,” *Appl. Organomet. Chem.*, vol. 33, no. 4, p. e4833, Apr. 2019, doi: 10.1002/AOC.4833.
